# Extracellular‐Matrix‐Reinforced Bioinks for 3D Bioprinting Human Tissue

**DOI:** 10.1002/adma.202005476

**Published:** 2020-12-09

**Authors:** Martina M. De Santis, Hani N. Alsafadi, Sinem Tas, Deniz A. Bölükbas, Sujeethkumar Prithiviraj, Iran A. N. Da Silva, Margareta Mittendorfer, Chiharu Ota, John Stegmayr, Fatima Daoud, Melanie Königshoff, Karl Swärd, Jeffery A. Wood, Manlio Tassieri, Paul E. Bourgine, Sandra Lindstedt, Sofie Mohlin, Darcy E. Wagner

**Affiliations:** ^1^ Lung Bioengineering and Regeneration Dept of Experimental Medical Sciences Stem Cell Centre Wallenberg Center for Molecular Medicine Lund University Lund 22362 Sweden; ^2^ Research Unit Lung Repair and Regeneration Helmholtz Zentrum München German Research Center for Environmental Health Ludwig‐Maximilians‐University University Hospital Grosshadern Member of the German Center of Lung Research (DZL) Munich 81377 Germany; ^3^ Laboratory for Cell Tissue and Organ Engineering Dept of Clinical Sciences Lund Stem Cell Centre Wallenberg Center for Molecular Medicine Lund University Lund 22362 Sweden; ^4^ Department of Experimental Medical Science Lund University Lund 22362 Sweden; ^5^ Soft Matter Fluidics and Interfaces MESA+ Institute for Nanotechnology University of Twente Enschede 7522 The Netherlands; ^6^ Division of Biomedical Engineering James Watt School of Engineering University of Glasgow Glasgow G12 8LT United Kingdom; ^7^ Dept of Cardiothoracic Surgery Heart and Lung Transplantation Wallenberg Center for Molecular Medicine Lund University Hospital Lund 22242 Sweden; ^8^ Division of Pediatrics Clinical Sciences Translational Cancer Research Lund University Cancer Center at Medicon Village Lund 22363 Sweden; ^9^ Present address: Department of Pediatrics Tohoku University Graduate School of Medicine Sendai Japan; ^10^ Present address: Division of Pulmonary Sciences and Critical Care Medicine Department of Medicine University of Colorado Denver Aurora CO USA

**Keywords:** 3D bioprinting, biofabrication, bioinks, extracellular matrix, tissue engineering

## Abstract

Recent advances in 3D bioprinting allow for generating intricate structures with dimensions relevant for human tissue, but suitable bioinks for producing translationally relevant tissue with complex geometries remain unidentified. Here, a tissue‐specific hybrid bioink is described, composed of a natural polymer, alginate, reinforced with extracellular matrix derived from decellularized tissue (rECM). rECM has rheological and gelation properties beneficial for 3D bioprinting while retaining biologically inductive properties supporting tissue maturation ex vivo and in vivo. These bioinks are shear thinning, resist cell sedimentation, improve viability of multiple cell types, and enhance mechanical stability in hydrogels derived from them. 3D printed constructs generated from rECM bioinks suppress the foreign body response, are pro‐angiogenic and support recipient‐derived de novo blood vessel formation across the entire graft thickness in a murine model of transplant immunosuppression. Their proof‐of‐principle for generating human tissue is demonstrated by 3D bioprinting human airways composed of regionally specified primary human airway epithelial progenitor and smooth muscle cells. Airway lumens remained patent with viable cells for one month in vitro with evidence of differentiation into mature epithelial cell types found in native human airways. rECM bioinks are a promising new approach for generating functional human tissue using 3D bioprinting.

## Graphical abstract

A new class of microscale phase‐separated hybrid bioinks is described by combining tissue specific extracellular matrix (ECM) with alginate. This novel rECM hybrid bioink is tissue specific, cytocompatible, biocompatible, and pro‐angiogenic, and is used to bioprint human airways composed of multiple, regionally specified primary human lung cell types.

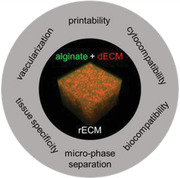

Recent advances in 3D bioprinting (i.e., 3D printing with cells) has generated enthusiasm for its potential for producing tissue for transplantation, but thus far, proof‐of‐concept studies have been limited to architecturally simple tissues, such as skin and cardiac patches.^[^ ^1^ ^]^ One of the main limiting factors has been a lack of bioinks, which simultaneously have properties needed for 3D bioprinting complex tissues as well as specific biological cues to support in vitro and in vivo tissue maturation.^[^ ^2^ ^]^ Several techniques have been explored to enhance biological activity of engineered materials and bioinks such as incorporation of specific ligands, individual extracellular matrix (ECM) components, or surface engineering of materials to enhance cell attachment and vascularization.^[^ ^3^ ^]^ However, these materials normally focus on enhancing the biological activity at one stage of tissue development (e.g., cell attachment or growth factors to promote vascularization). Multiple biological components and cues are needed in space and time to support the numerous steps needed to develop functional engineered tissue. Decellularized and solubilized extracellular matrix (dECM) derived from native tissue has emerged as a potential bioink with tissue‐specific composition.^[^ ^1^, ^4^ ^]^ However, their slow gelation kinetics limits the precision of constructs which can be generated and has severely hampered usage.^[^ ^2^, ^5^ ^]^ Therefore, structures generated using tissue‐specific dECM bioinks have been limited to simple grids when printed alone or require an external supporting structure (e.g., via thermoplastics) to retain the dECM solution during gelation.^[^ ^1^, ^4^ ^]^ Here we describe the development of a new class of tissue‐specific hybrid bioinks, which maintain biological activity during and after 3D bioprinting of complex and mechanically stable tissue. We show that this hybrid bioink system, composed of alginate reinforced with dECM (rECM) can be used to 3D bioprint perfusable tubes and branching structures at anatomically relevant length scales for human tissue without the need for an external support structure. Furthermore, the presence of ECM in the hybrid bioink system enhances survival of primary human progenitor cells during 3D bioprinting, supports tissue‐specific cellular differentiation, and stimulates full thickness vascularization of the implant in vivo while minimizing the foreign body response. As a proof‐of‐concept, we show that rECM bioinks containing lung dECM can be used to 3D bioprint a subsegmental human bronchus composed of regionally specified primary human lung smooth muscle and primary human airway epithelial progenitor cells which differentiate into multiple cell types found in human airways. Our work identifies rECM bioinks as a promising new class of bioinks for developing functional tissue for transplantation.

ECM solutions derived from pepsin‐digested decellularized tissues (Figure S1, Supporting Information) have been previously shown to form hydrogels when incubated at 37 °C due to spontaneous self‐assembly of ECM components, but require a support structure to form more complex geometries using 3D printing.^[^ ^1^, ^4^, ^5^, ^6^ ^]^ Additionally and similar to others, we found that not all ECM solutions can spontaneously form hydrogels, despite being processed similarly and retaining collagens (Figure S2, Supporting Information), such as collagen I and IV, at sizes known to be critical for hydrogel formation (Figure S2c,d, Supporting Information).^[^ ^5b^ ^]^ This indicates that dECM bioinks alone may not be suitable for 3D bioprinting of all tissues.

In order to obtain consistent and rapid gelation suitable for 3D bioprinting, we tested the potential of combining the ECM solution with another polymer commonly used in 3D bioprinting, alginate.^[^ ^1d^ ^]^ Alginate is regarded as non‐toxic and biologically inert to mammalian cells but does not contain any biological cues. However, one major advantage is that it can be quickly crosslinked with the addition of divalent cations to form hydrogels.^[^ ^7^ ^]^ We found that hydrogels could be rapidly formed from a hybrid mixture of alginate and ECM solutions upon Ca^2+^ addition (**Figure** **1**a). As rECM hydrogels were uniformly translucent, we examined the spatial distribution of individual components in crosslinked rECM hydrogels at higher resolution to determine whether they form interpenetrating networks or well‐mixed, phase separated hydrogels.^[^ ^8^ ^]^ For this, we generated rECM hydrogels using rhodamine‐labeled ECM solutions and fluorescein‐labeled alginate. While both components were retained within the rECM hydrogel, ECM components were well‐distributed in discrete foci, indicating the formation of a hydrogel with microscale phase‐separation (Figure 1b; Video S1, Supporting Information). We further confirmed microscale phase separation by performing scanning electron microscopy (SEM) under conditions which preferentially retained the alginate network and found that rECM hydrogels contained pores with larger sizes as compared to the alginate hydrogel at the same weight percentage (Figure 1c). As phase separated materials can be mechanically inferior to single phase materials, we examined mechanical properties at the bulk hydrogel level. Inclusion of ECM components in rECM hydrogels resulted in increased mechanical stability under shear stress compared to alginate hydrogels at the same weight percentage (Figure 1d; Figure S3, Supporting Information). Together, this confirms the formation of an alginate hydrogel network reinforced with dECM in its pores (rECM).

**Figure 1:**
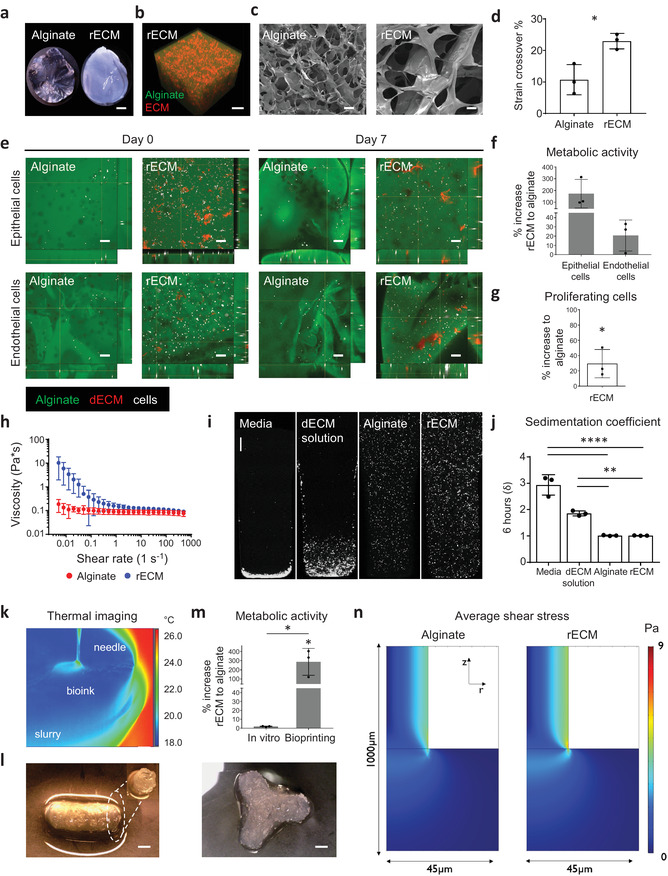
Characterization of rECM hybrid hydrogels. a) Picture of alginate and mouse rECM hydrogels. Scale bars: 1 mm. b) Alginate–fluorescein‐ and ECM–rhodamine‐modified rECM hydrogel showing the distribution of the alginate and ECM components within the hydrogel (see also Video S1 in the Supporting Information). Scale bar: 200 µm. c) SEM image of hydrogels. Scale bars: 50 µm. d) Strain crossover (%) between the storage and loss modulus in alginate hydrogels (2%) and rECM hydrogels (2% alginate, 5 mg mL^−1^ ECM) (*n* = 3 per group). e) Immunofluorescence images of murine lung epithelial MLE12 and endothelial bEnd3 (cells in white) in alginate–fluorescein (green) and ECM solution–rhodamine (red) modified rECM hydrogels on day 0 (day of seeding) and day 7. Scale bars: 100 µm f) Percent increase in metabolic activity of epithelial cells (MLE12) and endothelial (bEnd.3) cells in rECM hydrogels compared to alginate hydrogels on day 7 (*n* = 3 per group). g) Percent increase of EdU+ proliferating murine epithelial cells (MLE12) in rECM hydrogels compared to alginate hydrogels on day 5 (*n* = 3 per group). h) Oscillatory rheometry (*n* = 3 per group). i) Cell sedimentation confocal images and j) calculated sedimentation coefficient (δ) of A549 cells in DMEM–F12 cell culture media, alginate, mouse‐derived dECM and rECM solution for 6 h (*n* = 3 per group). Scale bar: 500 µm. k) Thermography of FRESH printing (see Video S2 in the Supporting Information). l) 3D bioprinted rECM hollow tube and branching structure (see Videos S3 and S4 in the Supporting Information). Scale bars: 2 mm. m) Metabolic activity (WST‐1 assay) on day 7 of seeded (in vitro) and 3D printed A549 cells in hydrogels (*n* = 3 per group). n) Average shear stress profiles of bioinks.

In order to test the cytocompatibility of the rECM bioink and resulting crosslinked hydrogels, we generated bioinks containing murine or human lung epithelial cell lines (MLE12 and A549, respectively) or a murine brain endothelial cell line (bEnd.3) labeled with cell tracker dyes and performed ionic crosslinking. Live cell imaging showed that cells proliferate in both alginate and rECM hydrogels, but cells grown in rECM hydrogels had enhanced metabolic activity over time (not statistically significant) (Figure 1e,f; Figure S4, Supporting Information). Increased metabolic activity corresponded to increased cell proliferation of murine lung epithelial cells in rECM hydrogels, as assessed by 5‐ethynyl‐2´‐deoxyuridine (EdU) staining and flow cytometry (Figure 1g), indicating that the ECM components are biologically active and induce proliferation.

We then characterized the rheological properties of rECM bioinks using oscillatory rheometry and found that the addition of ECM in the rECM solutions conferred shear thinning behavior as compared to alginate (Figure 1h), which is beneficial for 3D bioprinting.^[^ ^2^, ^7^ ^]^ Bioprinting of most clinically relevant products will take several hours, therefore bioinks, which prevent cell sedimentation are advantageous for mitigating clogging of the print head and generating larger constructs with homogenously distributed cells.^[^ ^2^, ^9^ ^]^ We found that bioinks containing alginate had significantly reduced cell sedimentation coefficients (δ) while ECM solutions had a δ similar to cell culture media (Figure 1i,j).^[^ ^2d^ ^]^ Therefore, both the alginate and the ECM components in rECM bioinks are required to simultaneously fulfil several optimum rheological and biological criteria for 3D bioprinting.

Next, we used freeform reversible embedding of suspended hydrogels (FRESH) 3D printing to generate 3D structures resembling anatomical structures (e.g., perfusable, hollow tubes and bifurcating structures representing blood vessels or airways) at relevant lengths for human tissue (i.e., mm–cm). Owing to the temperature difference, which exists between the bioink and support bath in FRESH 3D printing, we were able to visualize and monitor the 3D printing process in real‐time with thermography, which can be used for quality control of larger and complex constructs (Figure 1k–l; Figure S5a,b and Videos S2–S4, Supporting Information). Furthermore, we found that airways generated with rECM had increased yield strengths as compared to those generated with alginate alone, as assessed via myography (Figure S5c, Supporting Information). Thereafter, we investigated whether rECM bioinks support cellular viability during FRESH 3D bioprinting. We found that cells survived the printing process in both alginate and rECM derived hydrogels, with increases in cell numbers over seven days, indicating cytocompatibility of the process and subsequent hydrogel (Figure S5d, Supporting Information). Importantly, we observed that constructs retained their size and shape ex vivo, including open inner lumens, for up to 7 days. Cells, which were bioprinted in constructs using rECM bioinks had increased metabolic activity as compared to alginate (Figure 1m). Interestingly, changes in metabolic activity were not observed when hydrogels were formed using manual extrusion through a pipette (i.e., in vitro), indicating that the rECM bioink protects cells during 3D bioprinting, where cells are known to undergo increased shear stress (Figure 1m).^[^ ^2d^ ^]^ Therefore, we used computational fluid dynamics to investigate whether the rECM and alginate solutions have different fluid shear stress profiles leading to cell damage during the 3D printing process. Using the viscosities we previously determined experimentally (Figure 1h), we found that the average shear stress profiles for the two bioinks were highly similar (Figure 1n). This indicates that the difference observed in metabolic activity post‐printing does not originate from the bulk fluid properties but is likely due to the presence of biologically active factors within the ECM solution.

Vascularization and integration with the host is critical for the success of any transplanted tissue.^[^ ^10^ ^]^ However, 3D printing of capillaries is below the resolution limit of current 3D bioprinting techniques. Therefore, 3D printed constructs that are pro‐angiogenic are ideal to support short and long term graft survival. We used the chick chorioallantoic membrane (CAM) assay, a well‐established method to investigate angiogenic potential (**Figure** **2**a) and found that the rECM hydrogels promoted new vessel growth comparable to a material with known pro‐angiogenic properties (basement membrane extract (BME)).^[^ ^11^ ^]^ On the contrary, alginate hydrogels did not induce angiogenesis and acted similar to parafilm, a material with no known angiogenic properties (Figure 2b,c).

**Figure 2:**
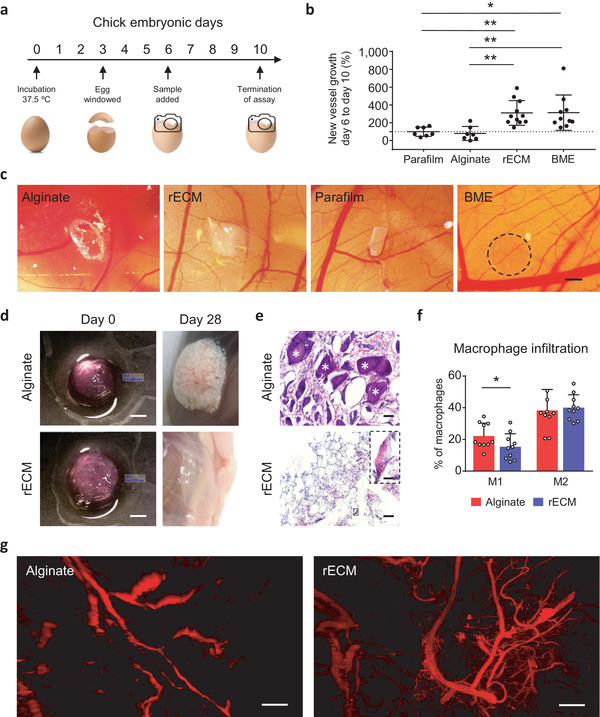
Biocompatibility and angiogenic potential of rECM hydrogels. a) Overview of CAM procedure. b) Changes in blood vessel formation, i.e., blood vessels on day 10 compared to day 6 and normalized to parafilm (100%) for each sample group. (*n* = 7–10 per group). c) Images of BME (positive control), parafilm (negative control), rECM and alginate hydrogels on CAMs on day 10. Scale bar: 1 mm. d) 3D printed alginate and rECM hydrogels in disk shape before subcutaneous implantation and when explanted on day 28. Scale bars: 2 mm. e) H&E staining of subcutaneously implanted alginate and rECM hydrogels after 28 days. White asterisks * indicate large, non‐proteinaceous debris. Inset showing red blood cells in the inner lumen of a blood vessel. Scale bars: 50 and 10 µm (inner panel). f) Macrophage infiltration (defined by CD45^+^, CD11b^+^, and F4/80^+^) in implanted alginate and rECM hydrogels on day 7 (*n* = 10 animals per group). g) Light sheet microscopy images (maximum intensity projections) of explanted alginate and rECM hydrogels (day 28) after optical clearing, showing blood vessel infiltration visualized by autofluorescence (*E*_x/_ *E*_m_: 480/520 nm) (see Videos S4 and S5 in the Supporting Information). Scale bars: 250 µm.

After initial angiogenic assessment in ovo, we investigated whether angiogenesis occurred in rECM hydrogels in vivo (Figure 2d). We subcutaneously implanted 3D printed hydrogels derived from alginate or rECM inks into T‐cell deficient *FoxN1* KO mice to mimic clinical immunosuppression in transplant patients.^[^ ^12^ ^]^ Initial assessment of explants on day 28 showed tissue encapsulation of alginate, whilst rECM hydrogels appeared well integrated into the surrounding tissue without any obvious signs of inflammation or foreign body response (Figure 2d). Histological analysis further confirmed major differences in tissue level remodeling between the two constructs, with large, non‐proteinaceous debris present in alginate hydrogels but not in rECM hydrogels (Figure 2e, white asterisks), characteristic of foreign body response observed with some forms of alginate.^[^ ^13^ ^]^ We next characterized the polarization of infiltrating macrophages, as this has been shown to correlate with remodeling outcomes of transplanted biological scaffolds (Figure S6 and Table S1, Supporting Information).^[^ ^13^, ^14^ ^]^ Anti‐inflammatory M2 macrophages were similarly present in both hydrogels after seven days whilst pro‐inflammatory M1 macrophages were increased in alginate as compared to rECM hydrogels (Figure 2f). This corresponds with previous work indicating the importance of suppressing the M1 phenotype for constructive remodeling.^[^ ^14^ ^]^ Additionally, we observed that a high proportion (≈80%) of infiltrating cells were CD45^−^ (Figure S6e, Supporting Information), which indicates the presence of non‐hematopoietic cells, such as endothelial and other stromal cells (e.g., fibroblasts and pericytes). In support of this, we observed blood vessels in both hydrogels which contained red blood cells, indicating connection with the host vasculature (Figure 2e), but the vasculature appeared more prevalent in the rECM constructs. Therefore, we examined the extent of vascularization throughout the entire construct via light sheet microscopy. rECM hydrogels supported the formation of an intact vascular network throughout the full thickness of the graft, composed of both large and small size blood vessels (Figure 2g; Figure S7 and Videos S5 and S6, Supporting Information). On the other hand, vasculature was less developed in the alginate hydrogel with evidence of deposits characteristic of the foreign body response throughout (Video S5, Supporting Information). Taken together, hydrogels derived from 3D printed rECM inks are biocompatible over 28 days, exhibit a lower inflammatory profile, and support neovascularization derived from the transplant recipient.

In order to move towards a proof of concept for using rECM bioinks for generating clinically relevant tissue, we tested whether rECM hydrogels containing ECM derived from lung tissue could support the growth and differentiation of primary epithelial progenitor cells isolated from normal human airways (Figure S8a, Supporting Information). Previous attempts to bioengineer airways have mostly focused on the use of decellularizing airways to obtain acellular scaffolds for subsequent recellularization, but attachment and differentiation of primary epithelial cells has been challenging due to degradation of ECM proteins.^[^ ^3c,d^ ^]^ We seeded human bronchial epithelial cells (HBECs), (KRT5+ and p63+ proximal airway progenitor cells) on hydrogels and grew them for 7 days in vitro to form a monolayer. Next, cells were lifted to air liquid interface (ALI) to mimic the tissue environment in vivo and cultured for up to one month (Figure S8b, Supporting Information). After 28 days, we observed that the cells had readily attached to the hydrogels and formed a multi‐layered epithelium in both alginate and rECM hydrogels (**Figure** **3**a). Cells at the basal side of the epithelium retained the expression of phenotypic markers characteristic of basal progenitor cells (KRT5+ and p63+), similar to human airways. We also observed evidence of differentiation towards multiple cell types present in human airways on the apical surface, such as mucin producing (i.e., MUC5AC+) and ciliated cells (i.e., Acetylated α‐tubulin+ and ciliated cells as observed by scanning electron microscopy) (Figure 3b,c). While the potential for differentiation towards ciliated cells was similar between the alginate and rECM hydrogels, we observed enhanced MUC5AC expression on the most apical layer of epithelial cells grown on rECM hydrogels (Figure 3b).

**Figure 3:**
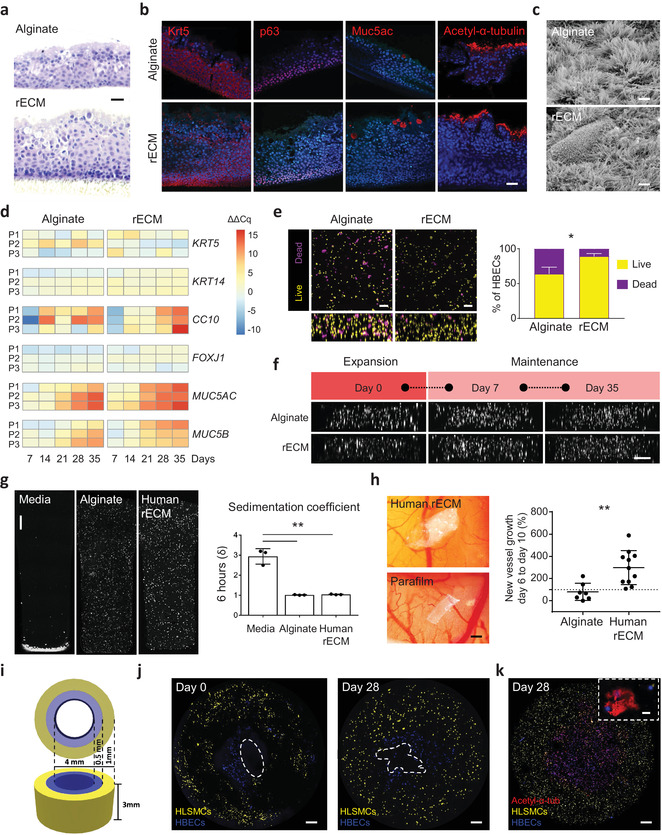
Human‐derived rECM hydrogels as bioinks for airways. a) H&E staining of HBECs 28 days after seeding on top of alginate and rECM hydrogels. Scale bar: 10 µm. b) Immunofluorescence stainings of KRT5, p63, MUC5AC and acetylated‐α‐tubulin positive cells 28 days after seeding HBECs on top of alginate and rECM hydrogels (*n* = 3 patients per group) Scale bar: 20 µm. c) SEM images of ciliated cells present in HBECs seeded on top of alginate and rECM hydrogels on day 28 (*n* = 3 patients per group). Scale bars: 3 µm. d) Changes in gene expression of HBECs seeded on top of alginate and rECM hydrogels and lifted to ALI on day 7 showing the fold change compared to day 0 of differentiation genes on day 7, 14, 21, 28, and 35 (increase in red and decrease in blue) (*n* = 3 patients per group (P1, P2, and P3)). e) Live–dead imaging and quantification of HBECs 3D bioprinted in alginate and rECM hydrogels right after bioprinting. The large panel is the top‐down view after seeding with side view below (live cells in yellow and dead cells in purple) (*n* = 3 patients per group). Scale bars: 100 µm. f) Side view of disk constructs with live cell tracker imaging of proliferating 3D bioprinted HBECS in alginate and rECM hydrogels on day 0, 7, and 35 (*n* = 3 patients per group). Scale bar: 200 µm. g) Cell sedimentation confocal images and calculated sedimentation coefficient (δ) of human lung epithelial A549 cells encapsulated in DMEM‐F12 media, alginate, and human derived rECM solution for six hours (*n* = 3 per group). Scale bar: 500 µm. h) CAM assay images of human‐derived rECM hydrogels and parafilm as negative control on day 10 and change in blood vessel on day 10 compared to day 6 normalized to parafilm in human‐derived rECM hydrogels (*n* = 7–10 per group). Scale bar: 1 mm. i) 3D rendering of bioprinted airways. j) 3D bioprinted airway with human lung smooth muscle cells (HLSMCs) in yellow in the outer perimeter and human bronchial epithelial cells (HBECs) in blue in the lumen on day 0 and on day 28 (*n* = 3 patients per group). Dotted white lines indicate the inner lumen. Scale bars: 500 µm. k) 3D bioprinted airway with acetylated‐α‐tubulin staining in red on day 28 (*n* = 3 patients per group). Inner lumen appears closed due to a processing artefact. Scale bar: 500 µm (lower) and 25 µm (upper).

We next examined a broader range of differentiation towards other cellular phenotypes found in adult human airways to see if the incorporation of tissue‐specific ECM can, in part, regulate differentiation of primary progenitor cells. Progenitor cells grown on rECM hydrogels containing ECM derived from lung tissue exhibited enhanced expression at earlier time points of multiple phenotypic markers found in the adult human airway epithelium as compared to alginate alone, indicative of differentiation ((e.g., ciliated cells (*FOXJ1*), goblet cells (*MUC5AC* and *MUC5B*), and club cells (*CC10*)) (Figure 3d; Table S2, Supporting Information). In addition, we observed a sustained increase in the pro‐regenerative airway phenotypic marker *KRT14* in all patients after lifting to ALI and consistent decreases in all patients for the basal cell marker *KRT5* on rECM.^[^ ^15^ ^]^ Finally, we tested whether primary HBECs survive 3D FRESH bioprinting, as primary cells are known to be more sensitive than cell lines. In line with our previous data (Figure 1m), we observed a significantly higher number of viable cells present in the rECM hydrogel (≈90%) as compared to alginate (≈60%) after bioprinting (Figure 3e) with homogenous distribution and viability up to 35 days (Figure 3f).

In order to establish a translationally relevant bioprinting workflow, which avoids the use of xenogeneic material, we sought to determine the feasibility of generating rECM hydrogels using decellularized human lung ECM. We batch‐processed lung ECM from four different decellularized human lungs (Figure S9a,b, Supporting Information) to reduce heterogeneity found in different patients and similarly generated rECM bioinks.^[^ ^16^ ^]^ We found that hydrogels formed after ionic crosslinking of human rECM and that hydrogels retained their translucent appearance (Figure S9c, Supporting Information), confirming the presence and retention of human lung ECM derived components. Similarly to murine rECM bioinks, human rECM bioinks prevented cell sedimentation for up to 6 h (Figure 3g) and were pro‐angiogenic in the CAM assay (Figure 3h).

Having confirmed that human rECM bioinks have beneficial rheological and biological attributes, we tested their ability for 3D bioprinting subsegmental bronchi, which are around 4 mm in diameter and composed of an outer smooth muscle layer and an inner epithelial layer.^[^ ^17^ ^]^ Subsegmental bronchi were generated by 3D bioprinting three concentric print layers: an inner layer of HBECs (blue) at a nominal diameter of 4 mm and two sequential layers of primary human lung smooth muscle cells (HLSMCs) (yellow) at nominal diameters of 5 and 6 mm, mimicking the anatomical location of the two cell types in human airways (Figure 3i). The dual extrusion system produced hollow tubes at high fidelity to the dimensions of the 3D digital model (Figure 3j; Figure S10a,b, Supporting Information) with distinct, but connected layers. Next, we cultured 3D bioprinted human airways for 7 days under submerged conditions, followed by 28 days at ALI of the entire construct (Figure S10c, Supporting Information). The inner lumen of 3D bioprinted human airways remained visibly open during ex vivo culture with no noticeable changes in dimensions, demonstrating that constructs are stable and patent for at least 28 days (Figure 3j, Day 28). Furthermore, cells remained within their respective layers of the engineered airways and HBECs differentiated into ciliated cells (positive for Acetylated α‐tubulin) (Figure 3k). Taken together, human rECM bioinks support the formation of a 3D bioprinted human airway composed of regionally specified primary human lung cells, which can differentiate towards mature human airway epithelial cell types.

Major advances have been made in generating shapes with increasing complexity using 3D bioprinting, but development of tissue‐specific bioinks compatible with these advances has remained an underexplored area. Recent reports have utilized ECM solutions from decellularized tissue as bioinks, but the long times needed for gelation has limited the precision and complexity of the shapes which can be generated.^[^ ^1^, ^5^ ^]^ Alternative approaches to accelerate gelation of hydrogels incorporating ECM have been explored by us and others using functionalized ECM for covalent crosslinking with synthetic polymers, but these functionalizations require harsh reaction conditions which degrade the ECM components and limit their biological activity.^[^ ^18^ ^]^ Other groups have used thermoplastics, such as polycaprolactone (PCL) to enable the use of 3D printing more complex shapes containing dECM, but cells cannot be printed within these thermoplastics and the two materials are mechanically weak at their interface.^[^ ^1^, ^4^ ^]^

In the present approach, we overcome the aforementioned limitations by generating tissue‐specific rECM hydrogels composed of alginate reinforced with dECM through microscale phase separation. We found that this combination of alginate and dECM is necessary to simultaneously fulfil several criteria for 3D bioprinting complex structures. The addition of the ECM to alginate in the rECM bioink results in shear thinning behaviour and the presence of alginate is necessary for resisting cell sedimentation. The alginate allows for rapid gelation upon ionic crosslinking while retaining the phase‐separated ECM in crosslinked hydrogels due to micro‐ and not macroscale phase separation. Microscale phase‐separation is ideal for forming hydrogels which retain both phases over time as we have found that the use of higher concentrations of ECM components in rECM can cause macroscale phase separation which leads to unstable constructs and rapid loss of rECM (data not shown). By controlling the ratio of alginate to dECM, we retain biological function over multiple stages of tissue maturation, including tissue‐specific differentiation of primary human progenitor cells, regulation of the immune response in vivo and vascularization upon transplantation. Finally, the rapid speed at which the alginate hydrogel network forms is ideal for generating 3D bioprinted constructs with complexity and precision at high fidelity to the 3D digital rendering. Protocols to decellularize tissue from almost every organ have been established and ECM solutions have been derived from a variety of different tissues and organs, therefore the current rECM system could be widely adapted for any tissue, including those ECM solutions that cannot spontaneously assemble on their own. However, bioreactors and ex vivo culture schemes for each tissue will need to be developed for precise control of factors (e.g., environmental, media, growth factors, etc.) that influence tissue maturation. Our work paves the way for the next generation of tissue‐specific bioinks and brings 3D bioprinting of human tissue for transplantation one step closer to reality.

## Conflict of Interest

The authors declare no conflict of interest.

## Supporting information

Supporting Information

Supplemental Video 1

Supplemental Video 2

Supplemental Video 3

Supplemental Video 4

Supplemental Video 5

Supplemental Video 6
