# Supplementary material for: Extracellular‐Matrix‐Reinforced Bioinks for 3D Bioprinting Human Tissue
Source: Adv Mater. 2020 Dec 9;33(3):2005476. doi: 10.1002/adma.202005476 (PMC11469085; doi:10.1002/adma.202005476)
Supplement: Supplementary file 1 — Supporting Information [file ADMA-33-2005476-s006.pdf]

© 2020 Wiley-VCH GmbH

ADVANCED MATERIALS

Supporting Information

for *Adv. Mater.*, DOI: 10.1002/adma.202005476

Extracellular-Matrix-Reinforced Bioinks for 3D Bioprinting Human Tissue

*Martina M. De Santis, Hani N. Alsafadi, Sinem Tas, Deniz A. Bölükbas, Sujeethkumar Prithiviraj, Iran A. N. Da Silva, Margareta Mittendorfer, Chiharu Ota, John Stegmayr, Fatima Daoud, Melanie Königshoff, Karl Swärd, Jeffery A. Wood, Manlio Tassieri, Paul E. Bourgine, Sandra Lindstedt, Sofie Mohlin, and Darcy E. Wagner**

Supporting Information

Reinforced Extracellular Matrix Bioinks for 3D Bioprinting Human Tissue

*Martina M. De Santis, Hani N. Alsafadi, Sinem Tas, Deniz A. Bölükbas, Sujeethkumar Prithiviraj, Iran A. N. Da Silva, Margareta Mittendorfer, Chiharu Ota, John Stegmayr, Fatima Daoud, Melanie Königshoff, Karl Swärd, Jeffery A. Wood, Manlio Tassieri, Paul E. Bourgine, Sandra Lindstedt, Sofie Mohlin, Darcy E. Wagner**

Experimental Section/Methods

Decellularization and derivation of ECM solutions: Mouse and human lungs were perfusion decellularized using a combination of detergent and enzyme solutions previously developed.[19] 8-14 week-old male and female wildtype C57BL/6J were obtained by Janvier and Charles River and housed in individually ventilated cages in rooms with constant humidity and temperature with 12-hours light cycle and access to water and food *ad libitum*. All animal studies were performed under the strict regulation of the Swedish board of agriculture and approved by the Malmö-Lund Animal Ethics Committee (Approval numbers: 5.8.18/12637/2017, M 152-14 and M 57-16). Human lung tissue was obtained from discarded surgical waste from donor lungs following lung transplantation. The study was approved by the Regional Ethics Review Board in Lund, Sweden (Dnr. 2017/396; Dnr 2018/386) and conducted in accordance with the Declaration of Helsinki with written informed consent from all patients and in accordance with the European Union General Data Protection Regulation (GDPR). Four human lungs were used for obtaining lung ECM. The average age of the patients was 36 years, with three females and one male.

ECM solutions were produced using slight modifications of a previously published protocol for deriving porcine lung ECM.[5a] Briefly, decellularized lungs were freeze dried (LABCONCO), milled into a powder using a TissueLyser (Qiagen) and digested using HCl

(0.1 M) and pepsin (1 mg µL^{-1}) (Sigma Aldrich) for 72 hours to generate an ECM solution (10 mg mL^{-1}). The pH of the ECM solution was increased using NaOH to deactivate the pepsin and the ECM solution was then neutralised to pH 7.4 ± 0.2.

DNA content: DNA content was assessed by using a Quant-iT™ PicoGreen™ dsDNA Assay following the manufacturer's instructions (Invitrogen™). DNA was extracted from murine and human native and acellular lungs and the absorbance measured with a Gen5 Microplate Reader (BioTek) (n=3-4 animals or patients).

Gelation Spectrometry: Gelation of ECM solutions at different concentrations (6, 8 and 10 mg mL^{-1}) and rat tail-derived collagen I (Corning) was assessed using spectrometry (n = 3/ condition). 200 µL of the solutions were pipetted into 96-well plates and absorbance measured at 450 nm at 37 °C every minute for 1 hour and normalized to PBS controls.

Preparation of hydrogels: Pre-gel solutions (i.e. bioinks) were made with either 2 wt% alginate in milliQ water (Sigma Aldrich, A0682) or for rECM solutions by combining 4 wt% alginate solution with ECM (10 mg mL^{-1}) solutions at a 1:1 ratio and mixing with a pipette to ensure a homogenous solution with final concentrations of 2 wt% alginate and 5 mg mL^{-1} ECM. Pre-gel solutions were crosslinked to form hydrogels with a $CaCl_2$ solution (50 mM).

Collagen quantification: Hydroxyproline content was quantified using the manufacturer's instructions (Hydroxyproline Assay Kit, Sigma Aldrich). All assays were measured using a PHERAstar FS spectrometer (BMG LABTECH) (n=3 animals and ECM solution batches).

Immunoblotting: Samples were normalized to tissue weight prepared in reducing conditions using β-mercaptoethanol (Saveen & Werner) and run on Tris-HCl gels followed by wet transfer onto polyvinylidene difluoride (PVDF) membranes (0.2 µm). 5% non-fat dry milk (BioRad) in Tris-buffered saline Tween-20 (TBST) was used for blocking and antibody dilutions. Primary

antibodies were incubated overnight at 4 °C, followed by addition of ECL clarity substrate (Bio-Rad) and imaging (ChemiDoc™ Touch, BioRad). Primary antibodies: Rabbit polyclonal anti-Collagen type I (600-401-103, Rockland Inc.; Dilution: 1:1000), rabbit polyclonal anti-Collagen IV (ab6586, Abcam; Dilution: 1:1000). Secondary Antibody: HRP Goat anti-Rabbit IgG (15015, Active Motif; Dilution: 1:3000).

Fluorescein labeled alginate: 1 wt% Na-alginate was dissolved in MES buffer (0.2 M) (Sigma Aldrich) at pH 5.5 with sodium chloride (0.6 M) (Sigma Aldrich). Alginate solution was mixed with Sulfo-NHS (N-hydroxysulfosuccinimde) (Sigma Aldrich) at 1:1 ratio to 1-Ethyl-3-(3-dimethylaminopropyl)carbodiimide (EDC) (Sigma Aldrich). To activate the carboxylic acids along the alginate polymer chain, EDC was added at a 1:10 molar ratio to uronic acid monomers of the alginate. Fluoresceinamine solution (4.5 mM) (Sigma Aldrich) was added 30 min later and left for overnight stirring. Fluorescein functionalized alginate was purified through dialysis using 12 kDa-14 kDa molecular weight cut-off (MWCO) cellulose membranes against NaCl (0.5 M) and DI water for 5 days.

Rhodamine labeled ECM: 0.5 w/v% ECM was dissolved in acetic acid (0.5 M) solution. Rhodamine B isothiocyanate (50 μg mL^{-1}) (Sigma Aldrich) was then added to the ECM solution and the reaction was performed at 4 °C for 48h. Rhodamine functionalized ECM was purified with 12kDa–14kDa MWCO cellulose membrane (Sigma Aldrich) through extensive dialysis against acetic acid (0.05 M) and then DI water for over a week. The product was lyophilized and stored at -20 °C.

Rheometry: Rheological measurements were carried out by using a stress-controlled rotational rheometer (MCR302, Anton Paar) equipped with different geometries depending on the measured samples. Pre-hydrogel solutions (~1 ml) were loaded between a cone and plate geometry (CP50-1) of 50 mm diameter and 1° angle for measuring their flow curves (Figure

1h). Hydrogels were loaded between two parallel plates (PP15) of 15 mm diameter and under a constant normal force and gap size, both ranging from 0.04 to 0.5 N and from 0.5 to 0.8 mm, depending on the samples, respectively.[20] Amplitude strain sweep tests were performed at a constant frequency of 10 rad/s and strain ranging from 0.1% to 100%.[21] All the measurements were performed at 25 °C (n=3 batches/condition).

Yield strength measurements: Measurements were performed by using a myography system (Danish Myo Technology). 3D FRESH printed alginate and rECM hydrogel rings (4 mm nominal outer diameter, single layer) were loaded onto the myograph and the passive force was measured as a function of distance starting from a distance of 1.5 mm between the wires (measured from the outer diameter of the wires) and measured in 0.2 mm increments, adjusted and monitored under the microscope. The yield strength was extracted from the passive force (mN) versus distance (mm) curves. All the measurements were performed at 25 °C in DMEM-F12 media (n=3-4 replicates/condition).

In vitro cell culture for cell lines and hydrogels: Murine and human lung epithelial cells (MLE12 and A549), murine brain endothelial cells (bEnd.3) and human lung smooth muscle cells (HLSMCs) were purchased from ATCC® (CRL-2110, CCL-185, CRL-2299 and PCS-130-010). Cell lines were cultured in DMEM/F12 medium (Gibco) supplemented with 10% FBS, streptomycin (100 mg L^{-1}), and penicillin (100 U mL^{-1}). Primary HLSMCs were cultured in vascular cell basal medium supplemented with vascular smooth muscle growth kit (ATCC®). Cells were grown using standard cell culture conditions at 37 °C, 5% CO_2 until 70–80% confluency and then mixed with pre-gel solutions (1 million cells/mL) to form bioinks. Bioinks were pipetted onto 24 or 96 well plates and crosslinked using a $CaCl_2$ solution (50 mM). Excess $CaCl_2$ solution was removed and DMEM/F12 medium added. Media was changed every other day.

Live staining was performed using the Cell Proliferation Staining Reagent Deep Red Fluorescence Cytopainter (abcam) following the manufacturer's instructions. Briefly, cells were incubated with the dye solution for 30 minutes, washed with PBS three times and then mixed in bioinks prior to crosslinking. Z-stack images were acquired using confocal microscopy (n=3/condition).

Confocal Microscopy: All samples imaged using confocal microscopy were imaged on a Nikon A1+ confocal system using a 10x or 20x objective.

Wst-1 assay: Metabolic activity was performed using Wst-1 (ab155902, Abcam, Cambridge, UK) following the manufacturer's instructions. Briefly, 20 µL of reconstituted Wst-1 reagent was added in 180 µL of media to 96 well plates containing cell laden hydrogels and incubated for 30 minutes at 37 °C, 5% CO_2. The absorbance was then measured at 490 nm using a PHERAstar FS spectrophotometer (BMG LABTECH).

Cell proliferation EdU assay: EdU-based proliferation was performed using Click-iT® EdU Alexa Fluor® 647 Imaging Kit (Invitrogen, Thermo Fischer Scientific) following the manufacturer's instructions with some modifications. Briefly, MLE12 cells were cultured for 5 days within the hydrogels (400K cells/mL hydrogel) and incubated for 24 hours with EdU solution, dissociated with EDTA (50 mM) in PBS for 10 minutes and fixed with 3.7% formaldehyde, and permeabilized with 0.5% TritonX-100. Alexa Fluor 647 secondary dye (Thermo Fischer Scientific) was used to visualize EdU positive cells. The number of EdU positive cells was measured and quantified by a FACS LSR II flow cytometer (BD Biosciences) (n=3 independent experiments/condition).

Cell sedimentation assay: Cell sedimentation within the different bioinks was assessed using a previously published assay.[2d] A549 cells were labeled with Cell Proliferation Staining Reagent Deep Red Fluorescence Cytopainter (abcam) following the manufacturer's

instructions and mixed to 1 million cells/mL in the different bioinks or DMEM/F12 media and murine ECM solution as controls. 70 µL cuvettes (BrandTech, 759220) were then filled with cell-laden bioinks, closed with parafilm and incubated at 37 °C, 5% CO_2 for 1 or 6 h. Samples were rotated 90° immediately prior to imaging using confocal microscopy along the entire height of the cuvette chamber (5 mm). Images were stitched together and divided into four vertical sections of equal size. Cells in each section were counted using ImageJ/Fiji v1.52p (Wayne Rasband, NIH, USA) and the cell sedimentation coefficient calculated as previously published (n=3 technical replicates/condition).[2d]

Freeform reversible embedding of suspended hydrogels (FRESH) printing: FRESH printing was performed as described previously with modifications.[1d] Briefly, a Replicator 2X Experimental 3D Printer (845-9567, MakerBot) was modified by replacing the plastic filament extruder with a 3D-printed syringe-based extruder. Bioprinting of individual bioinks (i.e. single extrusion) was done using the earlier model of the syringe extruder (3DPX-002102, NIH 3D Print Exchange), whereas bioprinting of two cell types was done with an updated model (Replistruder v3.0, YouMagine.com). A 2.5 mL Hamilton GASTIGHT 1000 series Syringe (26209, Sigma Aldrich) was mounted to the 3D-printed extruder equipped with a modified 27G needle (BD Microlance™). Pre-gel solutions were extruded into a 4°C cold 6% gelatin slurry containing $CaCl_2$ (20 mM) prepared as previously described.[1d] All 3D models were designed using Blender v2.79 (www.blender.org). STL files were exported from blender and post-processed using KISSlicer (www.kisslicer.com/) to generate the G-code at 50 µm-thick layers. Replicator G (replicat.org) or Simplify3D (www.simplify3d.com/) software were used to communicate the G-code to printer.

For single extrusion experiments, cells were fluorescently labelled with Cell Proliferation Staining Reagent Deep Red Fluorescence Cytopainter (Abcam) following the manufacturer’s instructions. The bioinks were prepared by mixing the stained cells with 2 wt%

alginate, mouse-rECM, or human-rECM solutions at a concentration of 10^6 cells/mL. Single extrusion was performed at approximately 0.14 µL s^{-1} and thermal imaging was done to monitor the 3D bioprinting process using a FLIR thermal camera (FLIR A655sc 25°, FLIR). For dual extrusion experiments, HBECs and HLSMCs were labeled respectively with Cell Proliferation Staining Reagent Blue and Deep Red Fluorescence Cytopainter (abcam) as above. Each bioink was separately prepared by mixing the stained cells with 2 wt% alginate or human-rECM solutions at a concentration of 10^6 cells/mL for HLSMCs and $3x10^6$ cells/mL for HBECs. 3D bioprinting of dual extrusion was done at a speed of 0.17 µL s^{-1}. Bioprinted 3D constructs remained in gelatin slurry, prepared as previously described, to crosslink for 30 minutes.[1d] The gelatin slurry was then dissolved at 37 °C. The 3D bioprinted airways (single or dual extrusion) were maintained in a 24 well, 0.4 µm pore size, transwell plates (Corning Costar). Constructs were lifted to ALI after 7 days by removing the expansion media (PneumaCult) and supplemented with ALI basal media (PneumaCult) in the outer chamber of the insert for 28 days with media changes every other day. Both HBECs and HLSMCs were in contact with air. Airway cross-sections were imaged using confocal microscopy (n=3 patients/condition).

3D Bioprinting simulations: Shear stress profiles during 3D printing were estimated by performing fluid dynamics simulations using the finite element method with COMSOL Multiphysics® 5.5 to model extrusion through the syringe. The system geometry was constructed as a 3.5 cm long needle (ID = 0.34 mm) and 3.5 cm of the syringe (ID = 7.35 mm) in polar coordinates (axisymmetric). To determine the velocity profiles, the Navier-Stokes equations were solved with a P2-P1 Lagrange elements (2nd order for velocity, 1st order for pressure). Simulations considered an inelastic response of the bioinks and used experimentally determined effective viscosity versus shear rate data as an input for rECM bioinks with a polynomial interpolation due to the complex rheological behaviour while for alginate a power-

law expression was found to be suitable to describe the data. The mesh consisted of 200,000 quadrilateral elements after successive refinement.

$$\rho\vec{u}\cdot\nabla\vec{u} = -\nabla p + \nabla\cdot\mu(\dot{\gamma})[(\nabla\vec{u}) + (\nabla\vec{u})^{\mathrm{T}}]$$

where ρ is the fluid density (kg/m^3), $\vec{u}$ is the velocity vector (m s^{-1}), p is the pressure (Pa), $\mu(\dot{\gamma})$ is the shear rate dependent effective viscosity (Pa·s) and $\dot{\gamma}$ is the shear rate (1 s^{-1}). The shear rate was estimated using the following equation:

$$\dot{\gamma} = \sqrt{\mathbf{2S}:\mathbf{S}} \text{ where } \mathbf{S} = \frac{1}{2}[(\nabla\vec{u}) + (\nabla\vec{u})^{\mathrm{T}}]$$

with a minimum value of 0.005 1 s^{-1} used for assessing viscosity, corresponding to the minimum value measured during rheological characterization of the inks.

The magnitude of the local average shear-stress was then determined by:

$$\tau = \mu(\dot{\gamma})\dot{\gamma}$$

Chick chorioallantoic membrane (CAM) assay: Fertilized eggs from Lohmann Brown chicken were commercially purchased and incubated in a BINDER incubator at 37.5 °C with constant humidity. A small window in the shell was opened on embryonic day 3 (E3) under aseptic conditions. The window was resealed with adhesive tape and eggs were returned to the incubator. On day E6, 10 µL of hydrogels (alginate, rECM hydrogel and BME (Cultrex®)) or parafilm were placed on top of the CAM. Eggs were resealed and returned to the incubator. On day E8, Ringer's salt solution was added on top of the CAM to prevent dehydration. Pictures were taken with a brightfield microscope (LEICA S9i) on E6 and E10 and analyzed with ImageJ/Fiji v1.52p (Wayne Rasband, NIH, USA) by three independent investigators in a blinded fashion (n=7-11/condition).

Subcutaneous Implantations: Alginate and mouse rECM disks (⌀: 6mm, h:2mm, print speed: 0.18 µL s^{-1}) were 3D printed as described above and subcutaneously implanted (6 disks/mouse) into *FoxN1* KO BALB/C background nude mice (animal ethics approval number: M15485-18). Macrophage response on day 7 was assessed using flow cytometry (LSRII/FORTESA) for the following markers: $CD45^+$, $CD11b^+$, $F4/80^+$, $CD11c^{+/-}$, $CD206^{+/-}$ (n=10 mice/condition) by pooling 3 constructs/condition from one mouse together and dissociating with EDTA (50 mM). Cell concentrations were then measured using an automatic cell counter (Countess™ II FL Automated Cell Counter, Invitrogen) followed by 1 hour incubation with primary antibodies. M1 macrophages were defined by $CD45^+$, $CD11b^+$, $F4/80^+$ and $CD11c^+$. M2 macrophages were defined by $CD45^+$, $CD11b^+$, $F4/80^+$, $CD11c^-$ and $CD206^+$. M0 (non-polarized) macrophages were defined by $CD45^+$, $CD11b^+$, $F4/80^+$ and $CD11c^-$. Details of antibodies used can be found in Table S1 of the supporting information. Implanted hydrogels on day 28 were explanted and fixed with 10% formalin for 4 hours (n=3 mice/condition).

Light-Sheet microscopy: Subcutaneously implanted hydrogels were processed using the iDISCO protocol as previously described.[22] Briefly, samples were washed with PBS and fixed at room temperature (RT) with 10% formalin for 1 hour. After extensive washing with PBS, the samples were dehydrated in a methanol/PBS series (20%, 40%, 60%, 80%, 100%, 100% for 1hr each), incubated in 66% dichloromethane / 33% Methanol for 3 hours, 100% dichloromethane for 30 minutes and in DiBenzyl Ether until before imaging. The samples were imaged submerged in dibenzyl ether using an Ultra Microscope II (LaVision Biotec) equipped with a 4x dipping objective (excitation/emission: 488/525 nm). 3D renditions and movies were created with Arivis Vision 4 D 3.1 (Arivis AG).

HBECS isolation: Human bronchial epithelial cells (HBECs) were isolated from discarded surgical waste from the bronchial anastomosis of donor lungs (Dnr. 2017/396; Dnr 2018/386) according to the Declaration of Helsinki and GDPR, with informed written consent. The

average age of the patients was 45, with two females and one male. Bronchi were dissected from the surrounding tissue and trimmed into smaller (1-2 cm^2) pieces and immersed in PBS with the addition of streptomycin (200 mg L^{-1}), penicillin (200 U mL^{-1}), Amphotericin B (5 µg mL^{-1}) and Gentamicin (100 µg mL^{-1}). Airway pieces were then incubated in 0.1% protease-DMEM/F12 media overnight at 4 °C and at 37 °C for 1 hour. Cells were isolated from the lumen by rinsing with DMEM/F12 media supplemented with 10% FBS and by rubbing gently on the walls with a pipette tip. The cell pellet was treated with DNase for 30 minutes at 37 °C, filtered through a 100 µm cell strainer and seeded in collagen I and collagen III (PureCol, Advanced Biomatrix) coated T75 flasks (~2 million cells/flask) for expansion with PneumaCult Expansion plus basal media supplemented with hydrocortisone (0.1 µg mL^{-1}), streptomycin (100 mg L^{-1}), penicillin (100 U mL^{-1}), Amphotericin B (2.50 µg mL^{-1}), and Gentamicin (50 µg mL^{-1}). Expansion media was changed every other day.

HBECS ALI culture and seeding onto hydrogels: ~80% confluent HBECs were lifted from the flasks and seeded onto alginate or rECM coated 24 well, 0.4 µm pore size, transwell plates (Corning Costar) at a 100,000 cells/insert. Cells were expanded with the antibiotic and hydrocortisone supplemented PneumaCult Expansion plus basal media until a confluent monolayer was formed and then lifted to ALI culture using 0.2 µg mL^{-1} heparin, 0.5 µg mL^{-1} hydrocortisone and antibiotic (streptomycin (100 mg L^{-1}), penicillin (100 U mL^{-1}), Amphotericin B (2.50 µg mL^{-1}), and Gentamicin (50 µg mL^{-1})) supplemented PneumaCult ALI basal media in the outer insert chamber up to 35 days. ALI media was changed every other day. Hydrogel coated inserts were fixed on day 28 with 10% formalin for 1 hour at RT (n=3 patients/condition).

Real-time RT-qPCR: RNA was extracted from snap-frozen hydrogels using 50 mM EDTA homogenization, TRIzol and chloroform extraction, following with RNeasy Micro Kit (Qiagen). The RNA was reverse-transcribed to cDNA using iScript™ Reverse Transcription

Supermix (BioRad), and then run in duplicate on a BioRad CFX96™ Real-Time system. Relative expression of *CC10, KRT5, KRT14, MUC5AC* and *MUC5B* (QuantiTect primers, Qiagen) was calculated as $2^{-\Delta Ct}$, with ΔCt calculated by subtracting the average Ct of *RPLP0* gene as a housekeeping control from the experimental sample Ct (n=3 patients/ condition).

Histological and immunofluorescence staining: Alginate and rECM samples were washed in PBS three times and OCT embedded in order to facilitate processing of 5 µm thin cryocuts, mounted on superfrost plus adhesion microscopic slides (Fisher Scientific), for histological and immunofluorescence staining. Haematoxylin and eosin staining was performed using standard methods and images were acquired on a Nikon H600L brightfield microscope. For immunofluorescence stainings, cryocuts were washed with PBS before being blocked for 1 hour with 5 wt% bovine serum albumin (Sigma Aldrich) in PBS, and incubated with primary antibody overnight at 4 °C. The following primary antibodies were used: Mouse anti-mucin 5ac (Muc5ac, ab3649, Abcam; 1:500), Rabbit anti-p63 (p63, ab124762, Abcam; 1:300), Rabbit anti-cytokeratin 5 (Krt5, ab52635, Abcam; 1:400). After incubation with appropriate secondary antibody for 1 hour, images were acquired using confocal microscopy (n=3/condition).

Scanning electron microscopy (SEM) imaging: Alginate or rECM hydrogels (without cells) were lyophilized without further crosslinking to preferentially preserve alginate networks (LABCONCO freeze drier). Samples were sputter coated with gold (with 40 mA current for 120 s) (Cressington, Watford, U.K) before being mounted and examined in a Jeol JSM-7800F FEG-SEM. For experiments where cells were seeded on top of hydrogels, samples were fixed in glutaraldehyde while in the coated inserts, followed by graded series of ethanol dehydration and critical point drying. Samples were sputter-coated with gold-palladium (with 40 mA current for 120 s) (Cressington, Watford, U.K) before being mounted and examined in a Jeol JSM-7800F FEG-SEM (n=3/condition).

Live Dead staining: Live Dead staining was performed on 3D bioprinted HBECS directly after the printing process (disks, as previously described for subcutaneous implants, from bioinks containing 1 million cells mL^{-1}). Calcein-AM (0.33 µM) (Sigma Aldrich) was used for live staining and propidium iodide (1 µg mL^{-1}) (Sigma Aldrich) was used for dead staining. 3 images per sample (3 samples per condition) were obtained using confocal microscopy. The number of live/dead cells in each image was quantified using ImageJ/Fiji v1.52p (Wayne Rasband, NIH, USA)).

Statistical analysis: Student t- tests were performed to compare the means of two normally distributed groups (alginate and rECM). Paired student t-test was performed to compare the means of alginate and rECM when the measurements were done using the same batch of alginate and the same cell passage number. One-way ANOVA was performed to test for statistical significance between the means of three or more normally distributed groups with a post hoc Tukey's multiple comparison test. Wilcoxon signed rank tests were used to assess significance between two non-normally distributed groups. In the case of graphs showing grow rates in percentages we tested the difference in mean between the conditions. All graphs show the mean and ± standard deviation (SD). Statistics were performed and graphs generated using GraphPad Prism 7 (GraphPad Software). P-values ≤ 0.05 are represented with (*), p-values $\leq$ 0.01 with (**), p-values ≤ 0.001 with (***) and p-values ≤ 0.0001 with (****).

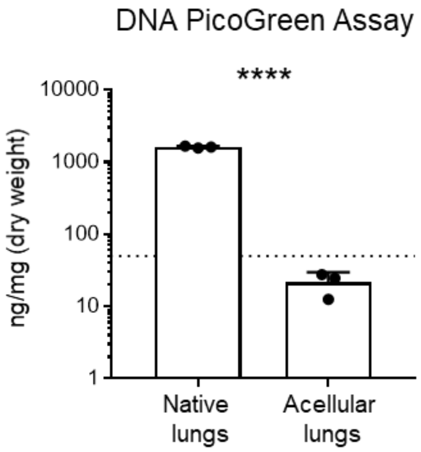

Figure S1: DNA quantification in native and acellular murine lungs using a PicoGreen assay. The dotted line marks the benchmark criteria of <50ng DNA/mg dry tissue weight for effective tissue decellularization (n=3 mice/group).[23]

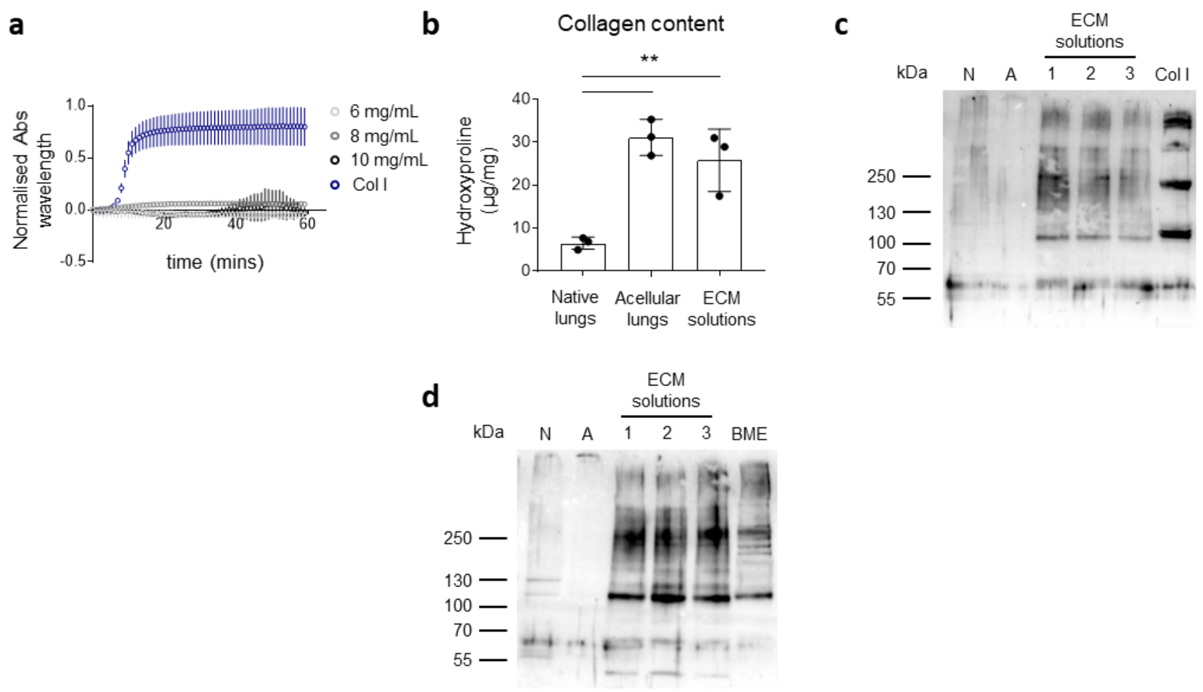

Figure S2: Mouse dECM solutions do not spontaneously gelate despite retention of collagens. a) Gelation kinetics of ECM solutions at different concentrations (6, 8 and 10 mg mL^{-1}) compared to rat tail type I collagen (3.7 mg mL^{-1}) (n=3/group). b) Hydroxyproline content in native lungs, acellular lungs, and ECM solutions (n=3 mice or batches/group). c) Collagen I Western blot showing collagen I content in native (N), acellular mice lungs (A), 10 mg mL^{-1} ECM solutions (1, 2 and 3) and rat tail type I collagen (Col I). d) Collagen IV Western blot showing collagen IV content in native (N), acellular mice lungs (A), 10 mg mL^{-1} ECM solutions (1, 2 and 3) and basement membrane extract (BME).

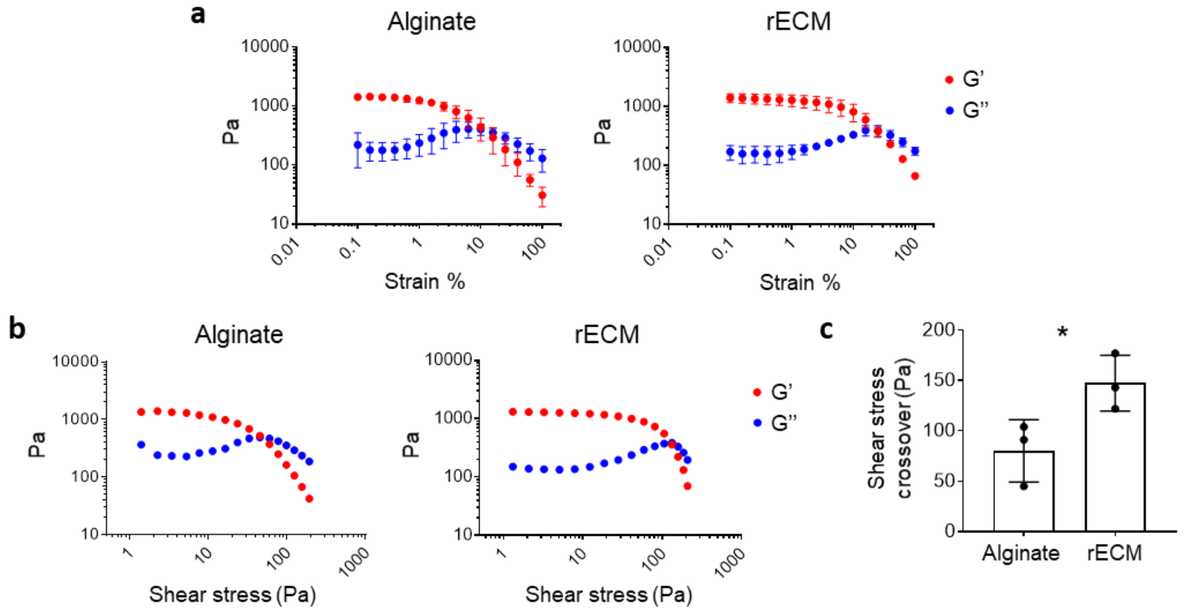

Figure S3: Mechanical testing of hydrogels. a) Mean curves of the moduli G' and G'' as a function of the strain (n=3 batches/group). b) Representative graphs of the moduli G' and G'' as a function of the shear stress (n=3 batches/group). c) Comparison between the shear stress crossover (Pa) of the moduli of alginate hydrogels (2%) and rECM hydrogels (2% alginate, 5 mg mL-1 ECM) (n=3 batches/group).

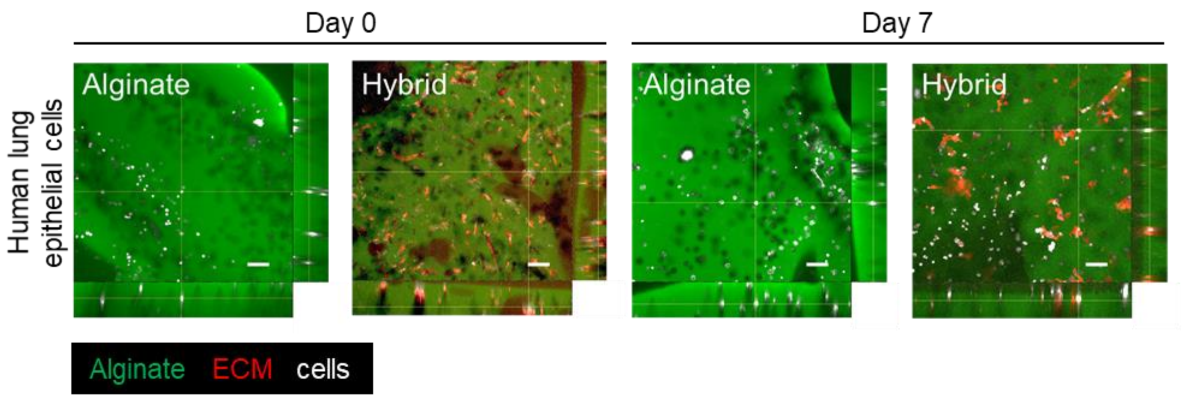

Figure S4: Immunofluorescence images of proliferating human lung epithelial A549 cells homogenously distributed in alginate-fluorescein and ECM solution-rhodamine modified rECM hydrogels on day 0 (day of seeding) and day 7. Alginate is labeled in green, ECM in red and the cells in white. Scale bars, 100 μm.

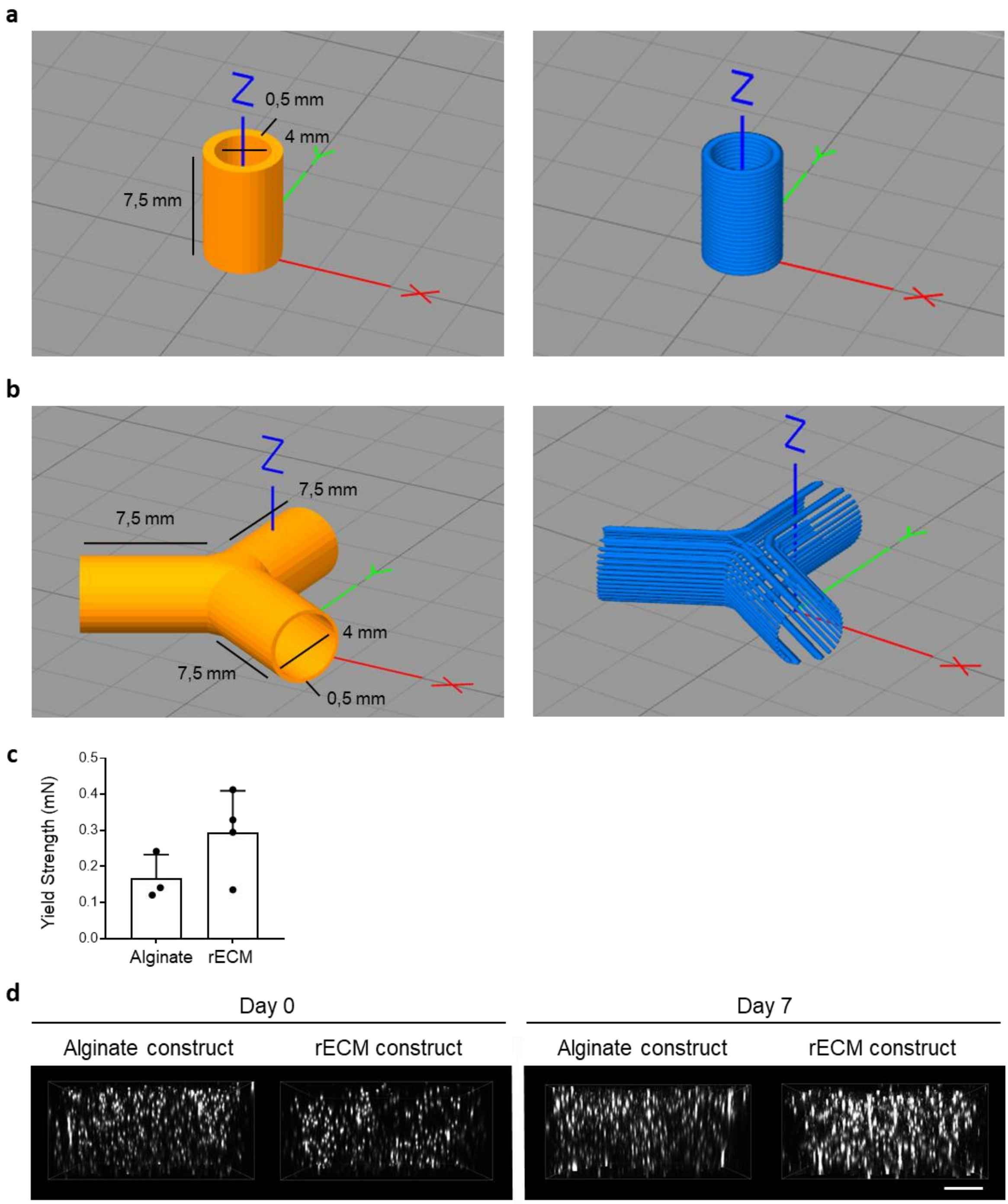

Figure S5: Digital rendering of bioprinted tube (a) and branching structure (b). Trace of extrusion movement shown in blue. c) Yield strength on 3D FRESH printed airway rings for alginate and rECM hydrogels as assessed by myography (n=3-4/group). d) Immunofluorescence images of proliferating A549 cells (white) in 3D bioprinted hollow alginate and rECM tubes on the day of printing (day 0) and day 7 (n=3). Scale bars, 200 µm.

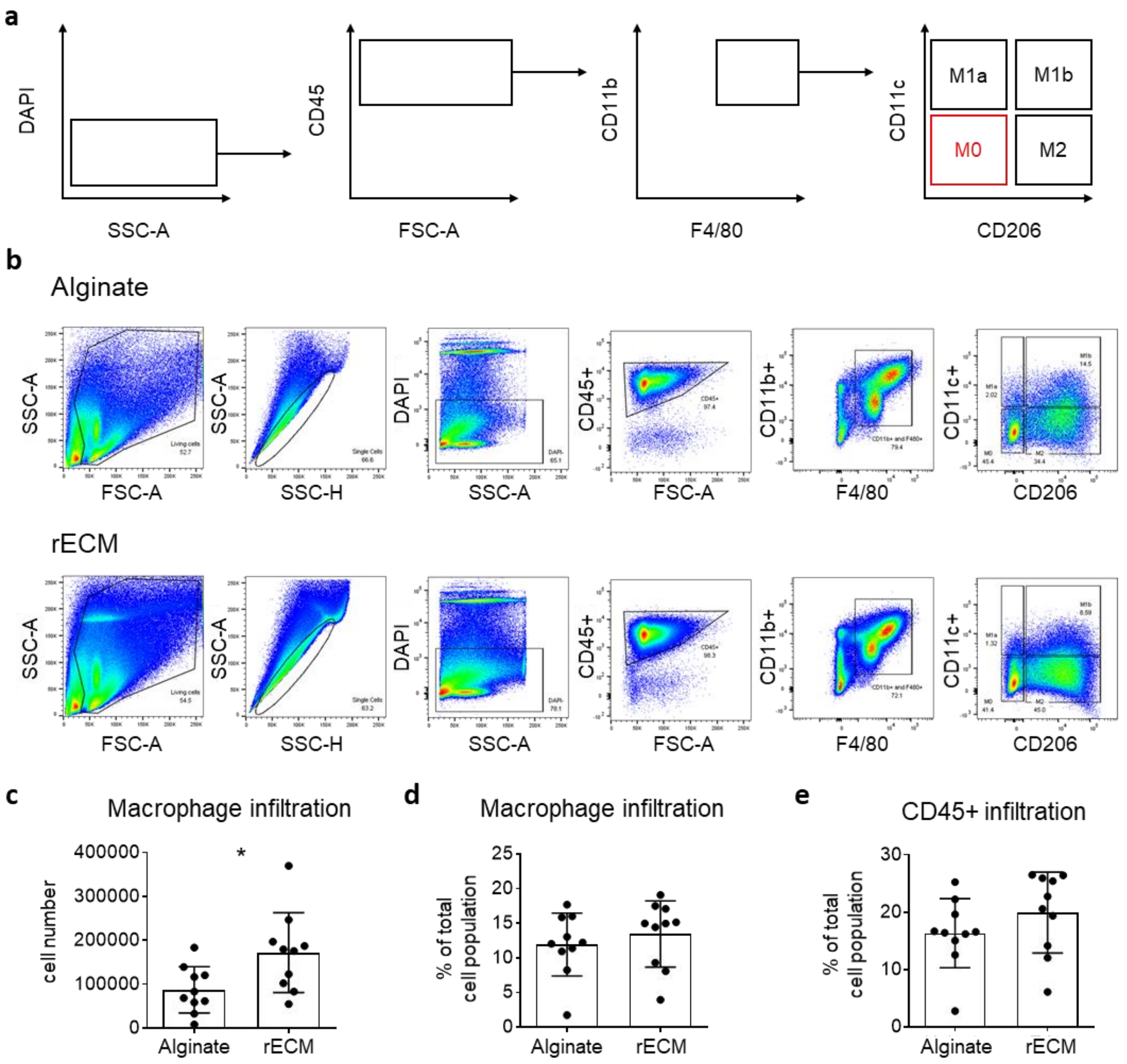

Figure S6: Macrophage infiltration in subcutaneously implanted alginate and rECM hydrogels. a) Flow cytometry gating strategy illustrating macrophage phenotyping. b) Representative flow cytometry gating of an explanted alginate and rECM hydrogel. c) Total macrophage infiltration in explanted alginate and rECM hydrogels (n=10 mice/group). d) Total macrophage infiltration in % of total cell population in explanted alginate and rECM hydrogels (n=10 mice/group). e) Total CD45+ cell infiltration in % of total cell population in explanted alginate and rECM hydrogels (n=10 mice/group).

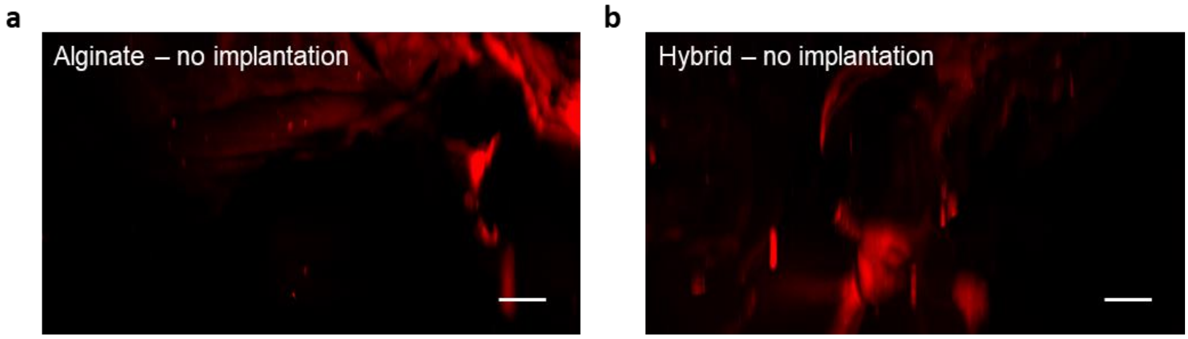

Figure S7: Light sheet microscope images (maximum intensity projections) of optically cleared alginate (a) and rECM hydrogels (b) not implanted, visualized by autofluorescence (Ex/Em: 480/520 nm) (see also Supplementary videos 5 and 6). Scale bars, 250 µm.

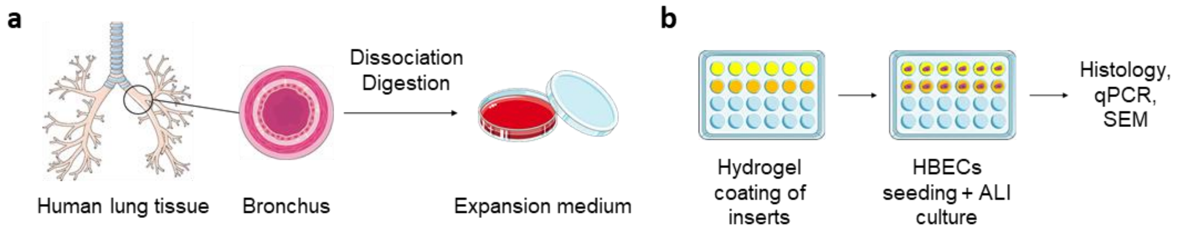

Fig. S8: Methodology of HBECs viability and differentiation on rECM hydrogels. a) Schematic for HBECs isolation protocol. Reproduced and modified from Servier Medical Art with permission. b) Schematic for HBECs culture on top of alginate and rECM hydrogels.

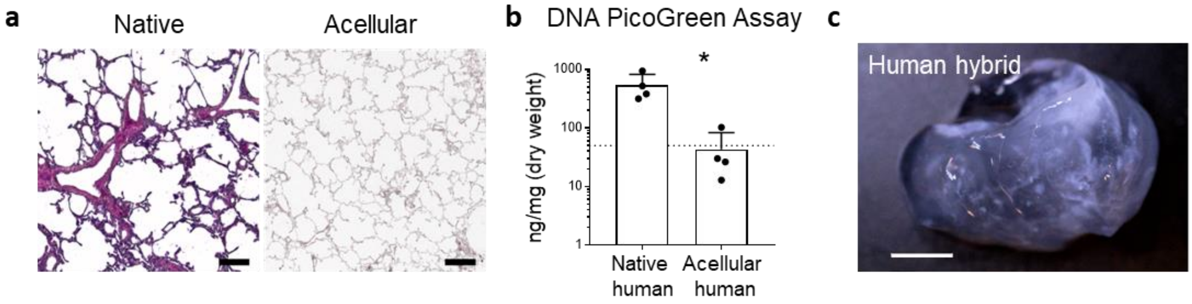

Figure S9: Human hybrid rECM hydrogels a) Representative H&E staining of human native and acellular lungs. Scale bars, 200 μm. b) DNA quantification in native and acellular human lungs using a PicoGreen assay. The dotted line marks the benchmark criteria of <50ng DNA/mg dry tissue weight for effective tissue decellularization (n=4 patients/group).[23] c) Picture of human hybrid rECM hydrogel. Scale bar, 2 mm.

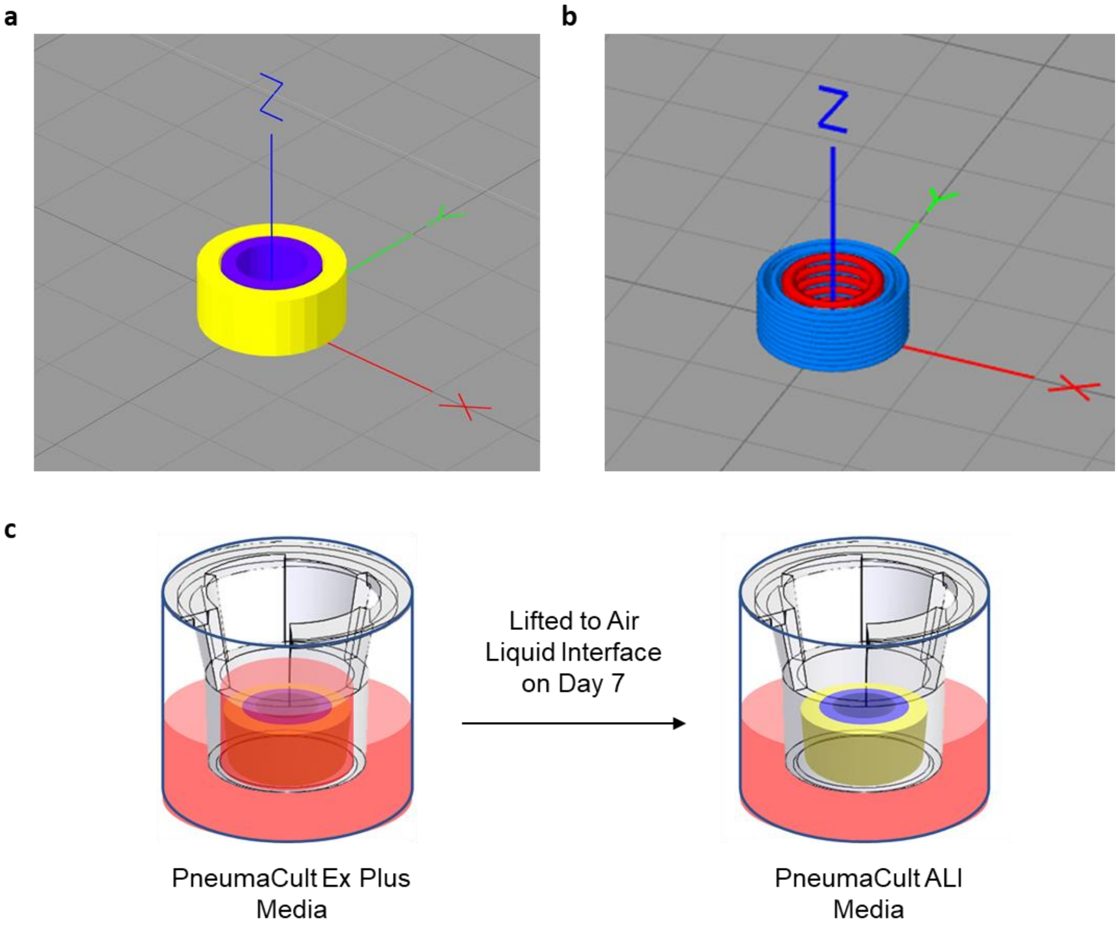

Figure S10: a) Digital rendering of bioprinted airways with human lung smooth muscle cells (HLSMCs) in yellow in the outer perimeter and human bronchial epithelial cells (HBECs) in blue in the lumen. b) Trace of extrusion movement when bioprinting. c) Schematic for culture of dual extrusion bioprinted airways in inserts at air liquid interface (ALI).

Table S1. Flow cytometry antibodies for subcutaneous implantations

Antibody	Cells	Dilution	Reference Biolegend
CD11b	Type I Macrophages, Monocytes, Dendritic cells, Granulocytes	1:75	101262
CD11c	Dendritic cells, Natural Killer cells	1:100	117318
CD206	Type II Macrophages	1:125	141712
CD45	Natural Killer cells, White Blood cells	1:200	147706
F4/80	Dendritic cells, Type I and II Macrophages	1:100	123110

Table S2. Changes in gene expression (ΔΔCq) of HBECs seeded on top of alginate and rECM hydrogels and lifted to ALI showing normalized gene expression per condition and patient over time. (n= 3 patients/group (P1, P2 and P3))

Sample	Timepoint (Day)	Patient	*KRT14*	*KRT5*	*FOXJ1*	*CC10*	*MUC5AC*	*MUC5B*
Alginate	7	1	-1.56	1.67	-2.80	-4.49	-1.98	-1.83
Alginate	7	2	1.03	3.71	0.95	-11.38	2.25	1.73
Alginate	7	3	0.22	0.90	0.77	-2.64	1.16	1.38
Alginate	14	1	-1.06	0.28	-1.19	8.65	3.51	-1.51
Alginate	14	2	1.43	7.77	-0.08	12.04	1.75	-0.20
Alginate	14	3	1.12	-3.18	-0.20	4.21	2.39	1.57
Alginate	21	1	-0.76	-1.02	-2.04	5.65	4.61	0.35
Alginate	21	2	1.50	3.16	0.38	2.44	7.75	3.88
Alginate	21	3	1.72	-2.06	0.10	1.71	8.88	5.42
Alginate	28	1	-0.28	3.72	-1.75	6.39	7.39	3.61
Alginate	28	2	2.28	8.53	0.30	12.47	11.49	7.84
Alginate	28	3	2.10	-0.30	1.31	-2.25	11.95	9.65
Alginate	35	1	-0.32	-0.95	-1.16	10.59	9.65	5.34
Alginate	35	2	2.21	5.24	0.63	11.37	13.05	9.17
Alginate	35	3	2.45	-0.57	1.49	6.47	13.31	10.35
rECM	7	1	-0.99	4.78	-0.80	-5.75	1.17	-0.49
rECM	7	2	3.09	0.76	1.67	-4.04	3.88	-1.52
rECM	7	3	3.14	6.03	-0.89	-3.19	5.48	0.71
rECM	14	1	1.44	6.95	-1.45	1.65	2.86	2.22
rECM	14	2	1.11	0.11	1.63	3.25	4.35	1.77
rECM	14	3	2.05	0.63	0.05	5.82	7.86	2.86
rECM	21	1	1.49	1.62	1.29	1.75	10.20	8.02
rECM	21	2	1.61	-1.74	1.53	2.41	9.81	6.09
rECM	21	3	2.19	-0.49	0.05	2.56	11.02	6.21
rECM	28	1	1.80	0.92	1.19	10.32	12.00	10.41
rECM	28	2	2.12	-1.60	1.75	8.73	12.24	8.54
rECM	28	3	3.92	4.06	-1.18	6.92	12.16	7.19
rECM	35	1	2.24	2.35	1.20	12.73	13.45	11.26
rECM	35	2	2.25	-2.14	1.48	11.40	12.79	8.57
rECM	35	3	3.09	-0.36	-0.95	16.38	14.00	8.10

Video 1: Alginate-fluorescein and ECM-rhodamine modified rECM hydrogel showing the distribution of the alginate and ECM components within the rECM hydrogel.

Video 2: Temperature based camera video of FRESH printing of an alginate solution into a $CaCl_2$ containing gelatin slurry using modified MakerBot printers.

Video 3: 3D bioprinted rECM hollow tube perfused with air.

Video 4: 3D bioprinted rECM branching structure perfused with air.

Video 5: Light sheet microscopy (maximum intensity projections) of optically cleared implanted alginate hydrogels (day 28), showing blood vessel infiltration visualized by autofluorescence (Ex/Em: 480/520 nm).

Video 6: Light sheet microscopy (maximum intensity projections) of optically cleared implanted rECM hydrogels (day 28), showing blood vessel infiltration visualized by autofluorescence (Ex/Em: 480/520 nm).
